# The Role of IL-10 in Malaria: A Double Edged Sword

**DOI:** 10.3389/fimmu.2019.00229

**Published:** 2019-02-12

**Authors:** Rajiv Kumar, Susanna Ng, Christian Engwerda

**Affiliations:** ^1^Department of Biochemistry, Institute of Science, Banaras Hindu University, Varanasi, India; ^2^Centre of Experimental Medicine and Surgery, Institute of Medical Sciences, Banaras Hindu University, Varanasi, India; ^3^Immunology and Infection Lab, QIMR Berghofer Medical Research Institute, Brisbane, QLD, Australia

**Keywords:** IL-10, malaria, protozoan, T cells, Inflammation

## Abstract

IL-10 produced by CD4^+^ T cells suppresses inflammation by inhibiting T cell functions and the upstream activities of antigen presenting cells (APCs). IL-10 was first identified in Th2 cells, but has since been described in IFNγ-producing Tbet^+^ Th1, FoxP3^+^ CD4^+^ regulatory T (Treg) and IL-17-producing CD4^+^ T (Th17) cells, as well as many innate and innate-like immune cell populations. IL-10 production by Th1 cells has emerged as an important mechanism to dampen inflammation in the face of intractable infection, including in African children with malaria. However, although these type I regulatory T (Tr1) cells protect tissue from inflammation, they may also promote disease by suppressing Th1 cell-mediated immunity, thereby allowing infection to persist. IL-10 produced by other immune cells during malaria can also influence disease outcome, but the full impact of this IL-10 production is still unclear. Together, the actions of this potent anti-inflammatory cytokine along with other immunoregulatory mechanisms that emerge following *Plasmodium* infection represent a potential hurdle for the development of immunity against malaria, whether naturally acquired or vaccine-induced. Recent advances in understanding how IL-10 production is initiated and regulated have revealed new opportunities for manipulating IL-10 for therapeutic advantage. In this review, we will summarize our current knowledge about IL-10 production during malaria and discuss its impact on disease outcome. We will highlight recent advances in our understanding about how IL-10 production by specific immune cell subsets is regulated and consider how this knowledge may be used in drug delivery and vaccination strategies to help eliminate malaria.

## Introduction

Malaria remains a major public health problem in tropical and sub-tropical regions of the world despite substantial efforts to reduce associated morbidity and mortality. There are still around 250 million cases and 500,000 deaths annually, with young children in sub-Saharan Africa being most affected (1). A major unmet medical need for malaria is an effective vaccine. No vaccine tested to date in malaria endemic areas has performed as well as when tested in healthy volunteers, including RTS,S/AS01 (2–4).

CD4^+^ T cells play critical roles in coordinating immune responses during infection by differentiating into functional subsets best suited to control pathogen growth (5). Diseases caused by intracellular protozoan parasites, such as *Plasmodium* species, require the generation of IFNγ-producing, Tbet^+^ CD4^+^ (Th1) cells to promote antigen capture and presentation by dendritic cells (DCs) and macrophages, as well as stimulate phagocytic cells to kill captured or resident pathogens (6). However, the inflammatory cytokines produced by Th1 cells can also damage tissues. In addition, recent data suggests that Th1 cell development may also influence the development of T follicular helper (Tfh) cells, another important CD4^+^ T cell subset in malaria needed for the expansion of antigen-specific B cell populations and the production anti-parasitic antibody (7, 8). Hence, a better understanding about the development of CD4^+^ T cell responses during malaria is needed to improve strategies aimed at improving anti-parasitic immunity.

The development of a robust host immune response is essential to eliminate parasites that cause malaria and protect against re-infection. Concurrently, these responses need to be tightly regulated to avoid immune-mediated damage to host tissue. This requires the establishment of immunoregulatory networks which ultimately determine the magnitude of immune response following infection. However, if these networks over-power anti-parasitic immunity too early, parasites can persist and cause associated disease. Many molecules and cell types contribute to these immunoregulatory networks, including anti-inflammatory cytokines such as interleukine-10 (IL-10) and transforming growth factor (TGF)β, immune check point molecules such as PD-1, CTLA-4, and LAG-3, as well as CD4^+^ FoxP3^+^ regulatory T (Treg) cells. However, our understanding about how immunoregulatory networks develop following *Plasmodium* infection and are maintained after resolution of infection is still incomplete.

One possible explanation for the failure of RTS,S/AS01 vaccine is the early imprinting of potent, pathogen-specific immunoregulatory networks in children following first exposure to malaria that prevents the generation of robust, vaccine-induced anti-parasitic immunity (9). Hence, targeting these networks may be critical step needed for malaria vaccines to stimulate long-lasting, anti-parasitic immunity in disease-endemic areas.

IL-10 has emerged as an important regulatory molecule in malaria that protects tissues by preventing excessive inflammation (10). It suppresses inflammation not only by directly dampening pro-inflammatory cytokine and/or chemokine production, but also by down-regulating the expression of MHC-II and co-stimulatory molecules on antigen presenting cells (APCs) and increasing expression of immune checkpoint molecules (11–13). IL-10 is secreted by many different cells, including B cells, Th1, Th2, Th17, and Treg cells, as well as innate immune cells such as macrophages and DCs (14). More recently, IL-10-producing Th1 (type 1 regulatory; Tr1) cells were found to develop relatively quickly in healthy volunteers participating in controlled human malaria infection (CHMI) studies and children living in malaria-endemic areas (15–18). Results from both pre-clinical malaria models and human studies show that IL-10 not only protects against severe disease, but also inhibits protective anti-parasitic immunity. In this review, we will discuss the role of IL-10 during the blood stage of experimental and human malaria, as well as describe the cellular sources of IL-10 and how the production of this potent anti-inflammatory cytokine is regulated. We will also examine how IL-10 mediated immune response may be manipulated to improve vaccine efficacy and/or current drug treatment regimes.

## IL-10 and Malaria

The balance between host pro- and anti-inflammatory immune responses plays a critical role in determining the outcome of *Plasmodium* infection. A weak pro-inflammatory response may result in uncontrolled replication of parasites, while an excessive pro-inflammatory response may cause tissue damage, such as occurs in severe malaria syndromes, including cerebral malaria and multi-organ failure. Studies from mice have identified a clear role of IL-10 in controlling inflammatory responses and preventing tissue damage (14). IL-10-deficient mice infected with *P. chaubadi chaubadi* AS displayed exacerbated disease pathology, including hypoglycemia, hypothermia, and a loss in body weight, along with enhanced pro-inflammatory cytokine (IFN-γ, TNF-α, and IL-12) production (19). The excessive pro-inflammatory conditions in *P. chabaudi* AS-infected IL-10-deficient mice were also thought to cause parasite sequestration to the brain, associated with cerebral edema and hemorrhages (20). IL-10 has also been reported to play a protective role in experimental cerebral malaria (ECM) caused by *P. berghei* ANKA (21). In this model, decreased expression of IL-10 mRNA in spleen and brain tissue was associated with susceptibility to ECM. Sequestration of parasitized RBC (pRBC) in brain was mediated by ICAM-1 expressed by endothelial cells, and IL-10 inhibited expression of ICAM-I on these cells, thus providing a potential mechanism for the prevention of pathology associated with ECM. In a lethal *P. yoelii* infection in mice, production of IL-10 and TGF-β were thought to inhibit pro-inflammatory responses, and this was correlated with high parasitemia and severe anemia (22, 23). IL-10 has also been reported to promote hyper-parasitemia in mice infected with *P. chabaudi adami* (24). Therefore, data from mouse models of malaria indicates that IL-10 is required to protect host tissue from inflammation, but by doing so, can also promote growth of parasites and associated disease manifestations.

In a prospective longitudinal study conducted in a malaria endemic area, *IL10* gene polymorphisms associated with high IL-10 production were found to increase the risk of developing clinical malaria in young children (25). High levels of circulating IL-10 have been reported in patients with mild, severe and cerebral malaria (26, 27). Similar to studies in pre-clinical models, African children with severe anemia had lower plasma IL-10 levels than patients with moderate anemia or cerebral malaria, suggesting that IL-10 plays an important role in preventing severe anemia (28). However, a case control study in an African population with mild or severe malaria showed that both IL-10 and TNF-α were elevated in severe malaria and positively correlated with parasitemia (29). In another study with children living in a holo-endemic area of Western Kenya, higher ratios of plasma IL-10 to TNF levels were strongly associated with protection against severe malaria anemia, providing evidence that IL-10 may be protective by inhibiting TNF activity (30). This was supported by data from pre-clinical malaria models that have shown over-expression of TNF can suppress haematopoiesis in the bone marrow and promote RBC destruction (31), while IL-10 is thought to enhance hematopoietic activity (32). High levels of plasma TNF have also been associated with anemia and high-density *P. falciparum* infection in Zairian children (33), as well as being associated with other severe malaria complications such as renal failure (34). Thus, IL-10 appears to play a critical role in regulating the pathogenic effects of TNF during malaria, but in performing this important role, IL-10 may promote high-density infections that can result in other complications of malaria, including accumulation of pRBC in tissue that can cause hypoxia and direct damage to the vasculature.

IL-10 suppressed IL-12 production by monocytes (35), which was required for the development of protective immunity against malaria and skewing the cytokine production pattern toward a pro-inflammatory response (36, 37). A study in patients with severe malaria anemia living in a holo-endemic region of western Kenya showed that ingestion of *Plasmodium*-derived pigment (hemozoin; [PfHz]) by monocytes, suppressed IL-12 production in an IL-10-dependent manner (38). IFNγ signaling is critical for the development of hematopoietic progenitor subsets during acute experimental malaria (39), and given the important role for IL-12 in IFNγ production, reduced levels of IL-12 would likely impact hematopoiesis during malaria.

IL-10 can also augment antibody production and B cell maturation (37, 40). In experimental malaria caused by *P. yoelli* infection of C57BL/6 mice, B cell intrinsic IL-10 signaling enhanced germinal center (GC) B cell responses by limiting IFNγ activity and subsequent Tbet expression by these cells, thereby promoting antibody production and parasite clearance (41). Hence, IL-10 may be beneficial for the development of humoral immunity, but detrimental for cell-mediated immune responses during malaria.

## Cellular Sources of IL-10 During Plasmodium Infection

IL-10 production was initially identified in Th2 cells (42), but has since been shown to be produced by many immune cells, including Th1 cells (43, 44), Treg cells (45), IL-17-producing CD4^+^ T (Th17) cells (46), Tfh cells (47), CD8^+^ T cells (48), B cells (49), including regulatory B cells (50), NK cells (51), and γδ T cells (52, 53). Additionally, innate immune cells such as macrophages and DCs (12) have also been shown to produce IL-10. In both lethal and non-lethal mouse models caused by *P. yoelii* infection, the major source of IL-10 were FoxP3-negative CD4^+^ T cells that didn't produce Th1, Th2, or Th17-associated cytokines, and these cells not only prevented hepatic immunopathology but also suppressed the effector T cell response, preventing parasite clearance (54). These regulatory cells are amongst several specialized CD4^+^ T cell sub-populations which emerge from the thymus as conventional CD4^+^ T cells and acquire regulatory functions in the periphery following exposure to inflammatory conditions (55–57). The most well-studied of these subsets are IL-10-producing Th1 (Tr1) cells that have been identified in many infectious diseases, including visceral leishmaniasis (58), tuberculosis (59), and human immunodeficiency virus (60). Importantly, they have also be identified as an important immunoregulatory cell population in African children with *P. falciparum* malaria (16–18, 61), as well as in healthy volunteers participating in CHMI studies (15). A high frequency of antigen-specific Tr1 cells were also found in neonates whose mothers had active placental malaria during pregnancy, suggesting that these cells might be able to influence anti-parasitic immunity from very early in life (62). Hence, the rapid generation of Tr1 cells during malaria may play a critical role in determining the outcome of infection. Furthermore, because these cells are likely to be generated prior to vaccination in malaria endemic areas, they are also likely to impact the efficacy of malaria vaccines, and their presence and function may be an important factor contributing to the failure of these vaccines to date.

γδ T cells play several different roles in host defense against *Plasmodium* infection (63), including their rapid expansion upon exposure to *P. falciparum* antigen in malaria naïve individuals (64). However, studies in children with chronic malaria exposure showed that the Vδ2^+^ subset of γδ T cells declined in number and switched from a predominant pro-inflammatory response to an anti-inflammatory response that was postulated to contribute to clinical tolerance during malaria (52). Another recent study in a malaria endemic area found a subset of γδ T cells from uncomplicated malaria patients expressing Vδ9 T cell receptor that expanded and produced IFNγ and IL-10 when cultured in presence of *P. falciparum* antigen (53). Given that these γδ T cells produce IL-10 and can provide help to B cells for antibody production (65), they may play a role in the acquisition of natural immunity against malaria.

NK cells have also been reported to produce IL-10 during many systemic infections (66, 67). A recent study in mice infected with *P. berghei ANKA* showed that systemic inflammation during ECM stimulated NK cell IL-10 production but was induced too late to prevent inflammation-mediated disease pathology (51). However, in the same study, treatment of mice with recombinant IL-15 complexed with antibody to extend cytokine half-life and target NK cell induced IL-10 production which protected against disease without affecting the parasite burden.

## Regulation of IL-10 by Cytokines

**Figure 1:**
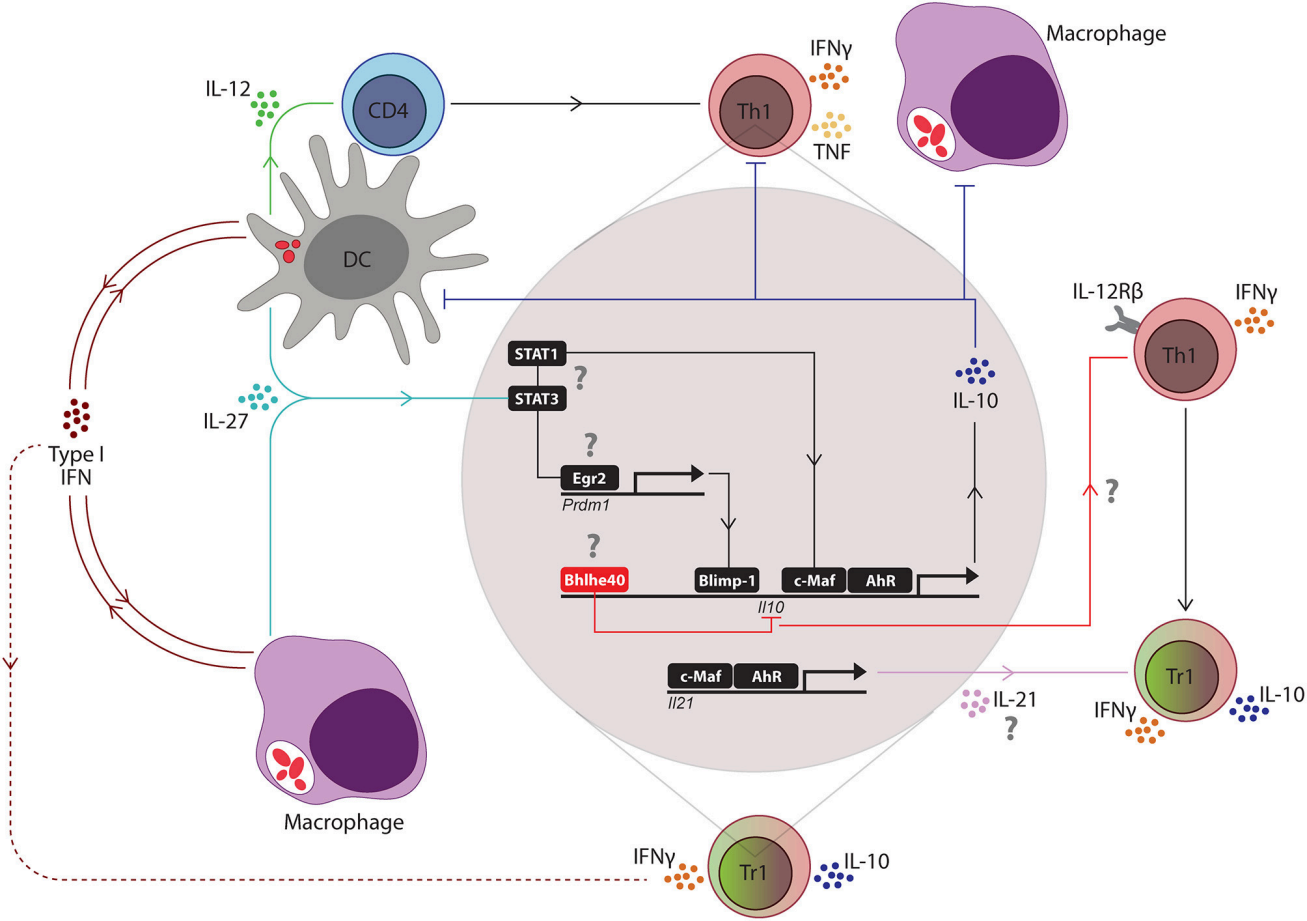
Key regulators of the Th1/Tr1 cell axis in mouse malaria models. Following blood-stage infection with *Plasmodium* parasites, dendritic cells (DC's) recognize parasite molecules and produce IL-12 to drive the expansion of antigen-specific CD4^+^ T cells into IFNγ-producing Th1 cells. Soon after, type I interferon (IFN) and IL-27 production is initiated by macrophages and DC's, presumably in response to activation of pattern recognition receptors by parasite molecules. Type I IFNs promote the development of IL-10-producing Th1 (Tr1) cells by T cell-independent and T cell-dependent activities (dashed red line), depending on the *Plasmodium* species. IL-27 stimulates STAT1 and STAT3-dependent transcription of c-Maf and aryl hydrocarbon receptor (AhR) in Th1 cells, which then drive *IL-10* and *IL-21* gene transcription. T cell receptor signaling of Th1 cells promotes expression of the transcription factor Erg2, which in turn, induces *Prdm1* (encoding Blimp1) transcription and IL-10-production by Th1 cells in a STAT3-dependent manner. IL-21 acts as an autocrine growth factor for IL-10-producing Th1 (Tr1) cells. IL-10 produced by these cells can suppress the activity of Th1 cells and phagocytes, as well as the antigen presenting capacity of DC's and macrophages. However, the Bhlhe40 transcription factor can also be upregulated in Th1 cells to block *IL-10* gene transcription and promote IFNγ production and IL-12 receptor β (IL-12Rβ) chain expression, which both re-enforce Th1 cell development and activity. Thus, Bhlhe40 has an important role in determining the balance between Th1 and Tr1 cell development. The small red ellipsoids in DC's and macrophages represent captured parasites and associated antigens. The question marks (?) indicate pathways not yet validated in *Plasmodium* infections.

Tr1 cells are an important immunoregulatory CD4^+^ T cell subset that not only prevent immune pathology during *Plasmodium* infection but can also promote establishment of infection by suppressing Th1 cell-mediated anti-parasitic immunity. The manipulation of this regulatory T cell subset is being considered for a wide range of immunotherapeutic applications, including in organ transplantation, rheumatoid arthritis, colitis, and cancer (68–71). Therefore, a better understanding about how IL-10 production is initiated and maintained by Tr1 cells is required for the development of new therapeutic approaches targeting this cell population. Tr1 cells are Tbet^+^ Foxp3^−^ CD4^+^ T cells that are most likely derived from Th1 cells, and acquire an ability to co-produce IL-10 and IFNγ under inflammatory conditions (72). IL-27 has been identified as a critical regulator of T cell IL-10 production in humans and mice (46, 73–76). Generally, IL-27 produced by APC's, such as DC's and macrophages, can induce expression of the transcription factors c-Maf and aryl hydrocarbon receptor (Ahr) via STAT1 and STAT3-dependent mechanisms, which in turn, stimulated IL-21 production by CD4^+^ T cells which acted in an autocrine manner to expand IL-10-producing Tr1 cells (77–79) (Figure 1). In mouse models of malaria, a critical role for IL-27 signaling in the regulation of pro-inflammatory Th1 cell responses and suppression of immune-mediated pathology has been reported (80–83). A study with *P. chaubadi* AS infection showed that although Treg cells produced IL-10 during infection, Tr1 cells were the important source of IL-10 for protection against severe immune-mediated pathology, and that although the generation of these Tr1 cells was dependent on IL-27 signaling, it was independent of IL-21 (84). Similarly, another study with mice infected with *P. yoelli* confirmed that Tr1 cell generation was dependent on IL-27 receptor signaling during blood stage malaria (85). Thus, IL-27 is a critical regulator of IL-10 producing Tr1 cells in mouse models of malaria. The involvement of IL-27 in Tr1 cell generation in human malaria has not been fully established, although patients with severe malaria were reported to have reduced IL-27 plasma levels, compared to uncomplicated malaria patients and endemic controls, suggesting a potential role in controlling pathology (86). Additionally, many of the intracellular signaling pathways described above and below (Figure 1) have yet to be confirmed in samples from experimental malaria or malaria patients. This is clearly an important gap in our knowledge. It should also be noted that Tr1 cells are likely to represent a heterogenous cell population (87), and cytokine production and responsiveness may be regulated distinctly, depending on local immune conditions.

More recently, type I interferons (IFNs) have emerged as important regulators of IL-10 production by Tr1 cells. Type I IFNs comprise a large family of cytokines that includes several types of IFNα and two types of IFNβ proteins which all signal through the common IFNα receptor (IFNAR) that consists of IFNAR1 and IFNAR2 chains which signal via STAT-1 and STAT-2 (88, 89) to mediate diverse functions during many infections (90). Polymorphisms in the *IFNAR1* gene have been associated with increased risk of severe malaria in The Gambia (91, 92), while a whole-brain transcriptional analysis in genetically resistant and susceptible inbred mice infected with *P. berghei* ANKA identified type I IFN-dependent transcriptional program associated with the pathogenesis of severe malaria ECM (93). Type I IFNs suppress CD4^+^ T cell-dependent parasite control during experimental blood-stage malaria by modulating the function of CD8α^−^ cDC following *P. berghei* ANKA infection, rather than acting directly on CD4^+^ T cells (94, 95). Another study in mice infected with *P. yoelli* YM showed that type I IFNs produced by plasmacytoid DCs (pDCs) activated conventional DCs (cDCs) and macrophages for generating B and T cell responses which are required for controlling parasitemia and mortality during late phase of infection (96). However, another study in mice infected with *P. yoelli* showed that type I IFNs directly promoted the expansion of Tr1 cells (97) (Figure 1). Significantly, in CHMI studies, type I IFNs produced by several different cell sources were found to be important regulators of developing anti-parasitic immunity. Type I IFNs not only suppressed innate immune cell function and parasitic-specific CD4^+^ T cell IFNγ production, but also promoted the development of parasitic-specific Tr1 cells (9). Thus, IL-27 is a major mediator of Tr1 cell development in mouse models of malaria, but to date, the main driver of Tr1 cell generation identified in humans infected with *P. falciparum* are type I IFNs. Whether these results point to separate pathways for Tr1 cell generation in humans and mice is still not clear, but if so, this has significant ramifications for developing strategies to modulate Tr1 cells for clinical advantage.

TNF is a potent pro-inflammatory cytokine that has been implicated in malaria pathogenesis. As mentioned above, the ratio of plasma IL-10 to TNF plays an important role in determining whether children with malaria develop anemia. It is often assumed that the relationship between these two cytokines is characterized by IL-10 dampening the pro-inflammatory activity of TNF. However, TNF can also promote IL-10 production, as demonstrated by TNF playing a major role in lipopolysaccharide-induced IL-10 secretion by human monocytes (98). Similarly, TGFβ was reported to play a protective role against severe malaria in mice (99), and TGFβ can drive IL-10 production by several different CD4^+^ T cell subsets, including Treg, Th17 and other FoxP3-negative cells (100). Therefore, modulation of IL-10 production in malaria may be achieved by targeting the activities of upstream activating cytokines, such as IL-27, IL-21, type I IFNs, TNF, or TGFβ. However, as indicated previously, the precise roles for these cytokines in clinical malaria still needs to be fully elucidated before these strategies can be developed and implemented.

## Transcriptional Regulation of IL-10

As discussed earlier, IL-10 can be produced by most CD4^+^ T cell subsets in various inflammatory settings, but whether IL-10 production is mediated by common transcription factors or cell lineage-specific transcription factors is unclear. Many transcription factors, including c-Maf, have been shown to modulate *IL10* gene expression *in vitro* (77, 101, 102). The ligand-activated transcription factor Aryl hydrocarbon Receptor (AhR) has also been shown to promote the development of Tr1 cells in humans (102). During IL-27-mediated Tr1 cell differentiation, AhR physically associates with c-Maf and trans-activates the *IL10* and *IL21* promoters (78) (Figure 1). A recent study in mice infected with *P. chabaudi* AS showed that c-Maf regulates T cell IL-10 production and T cell-specific c-Maf-deficiency was associated with greater acute-phase pathology, compared to control mice, but had little effect on blood parasitemia, similar to the phenotype observed in IL-10-deficient mice (103). Thus, although c-Maf-dependent T cell IL-10 production protected against the detrimental impact of inflammation, it had a minimal effect of the development of anti-parasitic immunity in this non-lethal, mouse malaria model.

Another transcriptional regulator, B lymphocyte induced maturation protein (BLIMP)1, has been shown to play an important role in IL-10 production by Treg cells (104). BLIMP1 is induced by IL-12 in a STAT4-dependent manner and was shown to control IL-10 expression by Tr1 cells in mice infected with *Toxoplasma gondii* (105). In chronic lymphocytic choriomeningitis viral infection, as well as in central nervous system-related autoimmunity, BLIMP1 was identified as a critical regulator of IL-10 production by Tr1 cells (106, 107). IL-27-dependent production of the early growth response gene 2 (Egr2), a transcription factor required for T cell anergy induction, was also required for IL-10 production by Tr1 cells in a BLIMP1-dependent manner (108, 109). BLIMP1-mediated IL-10 production by Tr1 cells was recently reported in experimental malaria (97), and BLIMP1-dependent IL-10 production by Tr1 cells protected against IFNγ-dependent, TNF-mediated splenic tissue damage, but also limited the control of *P. chabaudi* AS blood parasitemia (110).

More recently, basic helix-loop-helix family member e40 (Bhlhe40) has been identified as a negative transcriptional regulator of IL-10 production during *Mycobacterium tuberculosis* (111) and *T. gondii* infection (112). Interestingly, Bhlhe40 regulated IL-10 production in both T cells and DCs during *M. tuberculosis* infection by binding directly to the *Il10* gene promoter in both cell populations (111). In the case of *T. gondii* infection, Bhlhe40 promoted Tbet-dependent IFNγ production by CD4^+^ T cells, while also suppressing IL-10 production in this cell population (112). Given the many similar mechanisms of T cell IL-10 generation between these two infectious diseases and malaria, it will be important to establish the role of this transcription factor in IL-10 production during *Plasmodium* infections. Another basic helix-loop-helix family member Twist-1 also regulates IL-10 production (113), although little is currently known about its role in infectious diseases settings.

Therefore, key transcriptional regulators of IL-10 production have been identified with potentially important roles in malaria (Figure 1), although as mentioned previously, many still need to be validated with samples from pre-clinical models of malaria or malaria patients. However, this area of research is still at an early stage and it will be necessary to carefully elucidate the specific cell populations in which these transcription factors operate, the range of genes they regulate and whether their modulation can change IL-10 activity to improve anti-parasitic immunity without causing tissue damage.

## Therapeutic Manipulation of IL-10

IL-10 plays a critical role in the immunoregulatory networks that protect tissue from infection-mediated inflammation during malaria, and there is convincing mechanistic evidence form pre-clinical malaria models and associative data from malaria patient samples showing key roles for IL-10 is preventing several severe manifestations of malaria, including the development of anemia and damage to organs. These actions of IL-10 are like a double-edged sword, cutting both ways, as they can both suppress important anti-parasitic immune responses, and in particular, the functions of Th1 cell responses, but also protect the host from tissue damage. Furthermore, as mentioned above, IL-10 may also promote anti-parasitic antibody production by B cells. Therefore, given these beneficial and detrimental roles for IL-10 during malaria, it is critically important that we improve our understanding about how IL-10 production is regulated and the specific roles for IL-10 produced by different cell populations. We know that parasite-specific Tr1 cells develop early in children living in malaria endemic areas, as well as in healthy volunteers participating in CHMI studies with *P. falciparum*. However, given the important roles these cells play in preventing disease pathology, as outlined above, modulation of their activity to improve vaccine efficacy or the development of immunity after anti-parasitic drug treatment may be dangerous. However, if modulation of IL-10 production by specific cell populations or subsets can be achieved, leaving in place mechanisms to protect tissue from inflammatory mediators, then clinical benefits may be achieved. This will require the identification of unique cellular signaling pathways that regulate IL-10 production in different cell populations. In addition, strategies to target specific cell signaling pathways will need to be developed.

## Concluding Remarks

IL-10 is a critical immunoregulatory molecule with both positive and negative roles during malaria. Blocking IL-10 activity may promote anti-parasitic immunity by enhancing APC functions and associated T cell activity. However, this will likely result in concomitant tissue pathology and related disease. Therefore, a more selective strategy for IL-10 modulation will be needed. As we identify the cell subsets producing this cytokine, and learn more about upstream regulators, the cell signaling pathways, transcription factors and post-translational modifications controlling IL-10 production, we may be able to manipulate IL-10 activity to improve anti-parasitic immunity in response to vaccination or drug treatment. Although IL-10 is a promising target for immune modulation, it is not the only such target for trying to improve outcomes in malaria. Immune checkpoint molecules, other anti-inflammatory cytokines and alternative host-directed therapies have also been identified (9, 57, 114). Ideally, different approaches should be tested in parallel to establish the safest and best approach to take. However, robust pre-clinical and clinical models will be required, as well as appropriate resourcing. The development of effective, long-lasting immunity to malaria through vaccination or drug-mediated strategies is an important priority, and our increasing knowledge should help make this possible.

## Author Contributions

RK and CE researched the work and wrote the paper. SN prepared the figure. All authors reviewed and edited the manuscript.

### Conflict of Interest Statement

The authors declare that the research was conducted in the absence of any commercial or financial relationships that could be construed as a potential conflict of interest.
